# Artificial Intelligence in Dermatology Among Saudi Adults: Cross-Sectional Survey Study

**DOI:** 10.3390/healthcare14131963

**Published:** 2026-07-02

**Authors:** Shada Khalid Alanazi, Lama Nawaf Alanazi, Zahra Saleh Alsindi, Sarah Anwar Almulla, Nasser Abdulah Almulhim, Heba Yousef Al-Ojail

**Affiliations:** 1 College of Medicine, King Faisal University, Al-Ahsa 31982, Saudi Arabia; shatha19an@gmail.com (S.K.A.); anazilama@gmail.com (L.N.A.); zahralsindi@gmail.com (Z.S.A.); 2 Dermatology Resident, King Fahad University Hospital, Khobar 34445, Saudi Arabia; almulhimnasser115@gmail.com; 3 Department of Dermatology, College of Medicine, King Faisal University, Al-Ahsa 31982, Saudi Arabia; dr.heba.yousef@hotmail.com

**Keywords:** artificial intelligence, dermatology, public perception, teledermatology, cross-sectional study, Saudi Arabia

## Abstract

**Background/Objectives**: Artificial intelligence (AI) holds significant potential to enhance diagnostic support and access to dermatological care; however, its adoption depends on public trust and acceptance. This study aimed to assess knowledge, attitudes, and acceptance of dermatological AI among Saudi adults, and to identify factors associated with adoption, trust, and preferred system characteristics. **Methods**: A nationwide cross-sectional online survey was conducted among 668 Saudi adults (≥18 years) between 21 May and 5 June 2025, using convenience and snowball sampling via social media platforms (WhatsApp, Snapchat, Twitter/X, and Telegram). The questionnaire captured demographics, attitudes toward AI (20-item Likert scale), and perceived importance of six AI system features. Data were analyzed using descriptive statistics, one-way ANOVA, and binary logistic regression. The study was approved by the Institutional Review Board of King Faisal University (Approval No. KFU-REC-2025-MAY-ETHICS3443, approval date 19 May 2025). **Results**: The mean overall AI attitude orientation score was 74.48 ± 10.20 (Cronbach’s α = 0.868), reflecting moderately positive but conditional attitudes toward dermatological AI. Participants strongly preferred physician-supervised AI over fully autonomous systems, with medical oversight receiving the highest agreement (mean 4.27 ± 0.87). Privacy protection and diagnostic accuracy were rated as the most important system features. Age was significantly associated with the overall AI attitude orientation score (*p* = 0.009), with younger participants demonstrating more favorable orientations. Interest in technology showed the strongest association with both AI attitude orientation and perceived importance (*p* < 0.001). No demographic variable independently predicted high intention to use AI in multivariate analysis. **Conclusions:** Saudi adults generally exhibit favorable yet cautious attitudes toward dermatological AI. Implementation strategies should prioritize physician oversight, transparency, data privacy, and culturally responsive design to support responsible integration into clinical practice.

## 1. Introduction

Artificial intelligence (AI) has evolved from a speculative concept to an integral component of clinical dermatology. Deep learning systems, particularly convolutional neural networks (CNNs), have achieved diagnostic performance in melanoma detection and skin lesion classification that is comparable to board-certified dermatologists [1,2]. In addition to oncological applications, AI tools are increasingly utilized for disease severity scoring in psoriasis and atopic dermatitis, acne grading, alopecia pattern analysis, wound assessment, and dermoscopic interpretation [3].

Concurrently, large language model (LLM)-based systems have become a prominent category of AI utilized by dermatologists, primarily supporting treatment planning, patient education, and clinical documentation. An international survey across 13 countries found that LLM-based tools are the most frequently used AI modality in dermatology practice, with deployment patterns varying by disease context. Image-based systems are predominant in dermato-oncology, while LLM-based interfaces are more commonly employed in inflammatory and hair disorders [4]. Mobile applications and dedicated imaging platforms constitute additional implementation pathways, further diversifying the AI ecosystem in dermatology.

Despite these advances, clinical integration is constrained more by regulatory, ethical, and acceptance-related barriers than by technical capability [4]. Patient and public attitudes toward AI-assisted care are therefore central to successful implementation. Adoption depends not only on demonstrated accuracy but also on trust, perceived legitimacy, and willingness to engage with AI-mediated clinical pathways [5].

The current evidence on public acceptance of AI in dermatology is both limited and geographically concentrated. A recent narrative review of 48 studies on patient perspectives in dermatology and telemedicine found no data from Saudi Arabia or the broader Arab region [5]. Most existing studies have been conducted in Western and South Asian clinical settings, including US-based cohorts with relatively homogeneous demographics [6] and outpatient populations in Pakistan [7]. Recent research has identified race, skin tone, and familiarity with technology as key determinants of AI acceptance among the US population [8]. However, these findings are not readily generalizable to the Saudi context, where cultural, religious, and healthcare system factors differ substantially.

Saudi Arabia constitutes a distinct and underexamined context. AI integration has become a national healthcare priority, with increasing incorporation into administrative, analytical, and clinical workflows [4,9]. At the time of this study, dermatological AI tools were in limited but expanding use, primarily within teledermatology platforms and pilot image-analysis systems in academic centers. Consequently, the present cohort reflects a pre-adoption general population, providing a valuable baseline for understanding public attitudes prior to widespread implementation.

Cultural and ethical frameworks specific to Saudi Arabia may shape public perceptions of AI in healthcare. Islamic bioethical principles emphasize values such as privacy, human dignity, accountability, and professional responsibility, which may influence attitudes toward AI-assisted medical decision-making. In addition, cultural and religious norms related to modesty and bodily privacy may affect acceptance of image-based dermatologic technologies [10,11]. These dimensions are not represented in current evidence. In addition to this geographic gap, a cross-stakeholder gap remains. While recent studies have characterized clinician perspectives on AI in dermatology, including international data and Saudi-specific surveys [4,12], public attitudes have not been systematically evaluated. This limitation restricts the development of aligned, patient-centered implementation strategies.

This study addresses three key gaps: (1) the absence of dermatology-specific AI acceptance data from the Saudi general population; (2) the lack of culturally grounded frameworks that incorporate local ethical and social considerations; and (3) the absence of public-facing data to complement existing clinician-focused evidence. The objectives of this study are to characterize knowledge, attitudes, and acceptance of AI in dermatological care among Saudi adults, to identify factors associated with acceptance, and to provide the first population-level data from Saudi Arabia to inform context-specific implementation of AI in dermatology.

## 2. Methods

### 2.1. Study Design and Participants

This nationwide cross-sectional study included 668 Saudi adults and was conducted between 21 May and 5 June 2025. Participants were recruited using convenience sampling via social media platforms (WhatsApp, Snapchat, X [formerly Twitter], and Telegram). Snowball sampling was additionally employed, with participants encouraged to share the survey link within their networks to enhance geographic representation across Saudi Arabia.

Eligible participants were Saudi nationals aged ≥18 years who were able to read Arabic and had internet access. Individuals younger than 18 years, non-Saudi residents, or those unable to complete the survey were excluded.

### 2.2. Sample Size

The minimum required sample size was calculated as 384 participants using the Cochran formula, assuming a 50% expected prevalence of positive attitudes toward AI, a 5% margin of error, and a 95% confidence level. After accounting for a 15% non-response rate, the target sample size was increased to 442 participants. The final sample comprised 668 participants, exceeding the required sample size and providing adequate statistical power for the analyses performed.

### 2.3. Survey Development and Validation

The study instrument comprised three sections: (1) sociodemographic and technology-related characteristics, (2) attitudes toward artificial intelligence (AI) in dermatology, and (3) perceived importance of key AI system features.

Item development was informed by the framework of Wu et al. [6]. Its structure was informed by established digital health acceptance frameworks, including the Technology Acceptance Model [13,14], and encompassed six subdomains: knowledge, trust, perceived benefits, cultural considerations, clinical concerns, and intention to use. A separate domain assessed the perceived importance of AI system characteristics.

The final attitude scale included 20 items rated on a 5-point Likert scale (1 = strongly disagree to 5 = strongly agree). All items were scored in their original direction, and a total composite score was calculated by summing item responses (range: 20–100). Higher scores indicate a more favorable overall orientation toward dermatological AI. The composite score was calculated using the original response directions of all 20 items, including concern-oriented items on privacy, treatment delays, algorithmic bias, physician preference, and medical supervision. Consequently, the composite score reflects a multidimensional attitudinal orientation rather than a unidimensional measure of acceptance. To support interpretation, item-level and domain-specific results are presented alongside the aggregate score. The third section evaluated six core AI system features: data privacy and security, diagnostic accuracy, interpretability, cultural and religious compatibility, ease of use, and cost. These were rated on a 5-point scale ranging from 1 (not important) to 5 (very important).

Content and face validity were established through expert review (n = 5). Items were assessed for clarity, clinical relevance, cultural appropriateness, and domain alignment, and were iteratively refined until consensus was achieved. Linguistic validation was performed using a forward–backward translation process. Discrepancies were resolved by consensus to ensure semantic equivalence.

A pilot study involving 50 participants confirmed clarity and usability, with acceptable internal consistency (Cronbach’s α = 0.834). In the main study, the overall scale demonstrated excellent reliability (Cronbach’s α = 0.868; McDonald’s ω = 0.888). Subdomain-level internal consistency varied (perceived benefits α = 0.792; intention to use α = 0.849; cultural considerations α = 0.690; clinical concerns α = 0.575; trust α = 0.514; knowledge α = 0.465), reflecting the limited number of items per domain. Accordingly, subdomain scores were interpreted descriptively rather than as independent latent constructs.

### 2.4. Data Collection and Storage

The validated survey was administered electronically via Google Forms (Google LLC, Mountain View, CA, USA) with mandatory responses, yielding a complete dataset of 668 responses with no missing values. Data were stored in encrypted, password-protected, access-restricted institutional systems.

### 2.5. Data Analysis

Statistical analyses were performed using IBM SPSS Statistics version 28.0 (IBM Corp., Armonk, NY, USA). Categorical variables are presented as frequencies and percentages, whereas continuous variables are reported as means ± standard deviations (SD).

Internal consistency of the composite attitude scale and its subdomains was assessed using Cronbach’s alpha (α) and McDonald’s omega (ω). The composite attitude score was calculated by summing responses across all 20 Likert-scale items (range: 20–100), with higher scores indicating a more favorable overall orientation toward dermatological AI. Subdomain scores were calculated as the sum of their constituent items. A composite perceived importance score was similarly derived by summing responses across the six AI system-feature items (range: 6–30).

Differences in attitude and perceived importance scores across demographic and technology-related characteristics were evaluated using one-way analysis of variance (ANOVA). Binary logistic regression was performed to identify factors independently associated with a high intention to use dermatological AI. High intention was defined as a mean score ≥ 4.0 on the two-item intention subdomain (equivalent to a total score ≥ 8 on a scale ranging from 2 to 10). All demographic and technology-related variables were entered simultaneously into the model. Results are reported as odds ratios (ORs) with 95% confidence intervals (CIs). Statistical significance was defined as a two-sided *p* value < 0.05.

### 2.6. Ethical Considerations

The study was conducted in accordance with the Declaration of Helsinki and approved by the Institutional Review Board of King Faisal University (Approval No. KFU-REC-2025-MAY-ETHICS3443). Electronic informed consent was obtained from all participants prior to survey access. No personally identifiable information was collected, and all data were stored securely.

## 3. Results

### 3.1. Participant Characteristics

A total of 668 participants were included. Most respondents were aged 18–24 years (45.4%), followed by 25–34 years (28.0%), and 61.1% were female. Participants were primarily from the Central (44.2%) and Eastern (28.3%) regions. Over half held a bachelor’s degree (55.5%), while 24.7% had a high school education or less.

Regarding dermatologic care, 37.0% had visited a dermatologist multiple times, whereas 26.8% had never done so. Most participants (73.1%) were not affiliated with the healthcare field. Technological proficiency was generally moderate (70.8%), with 33.5% reporting high interest in new technologies. More than half (55.7%) reported using mobile health applications (Table 1).

### 3.2. Attitudes Toward AI in Dermatology

Item-level responses are summarized in Table 2. Findings are presented according to whether items reflected positive perceptions of AI, preferences regarding oversight and governance, or concerns related to AI use.

Items reflecting positive perceptions of AI showed generally favorable attitudes. Participants reported a positive outlook toward AI in healthcare (3.91 ± 0.93) and recognized its potential to improve access to care in remote areas (3.92 ± 0.94), reduce healthcare costs (3.81 ± 0.86), and support early detection of serious skin diseases (3.67 ± 0.95). Trust in AI for skin diagnosis (3.26 ± 0.97) and in emergency situations (3.35 ± 1.03) was comparatively lower.

The highest levels of agreement were observed for items related to oversight and governance. Participants strongly agreed that AI should operate under medical supervision (4.27 ± 0.87), that AI-generated decisions should be explainable (4.12 ± 0.82), and that physician judgment should take precedence when AI and physicians disagree (4.11 ± 0.86). Participants also expressed strong trust in dermatologists’ expertise (3.96 ± 0.83) and agreed that AI systems should respect cultural values (3.97 ± 0.90).

Several items reflected concerns regarding AI use. Participants reported concerns about treatment delays (3.94 ± 0.91), privacy and data security (3.80 ± 1.01), and potential algorithmic bias (3.23 ± 1.16). There was also substantial agreement with the statement that additional information about AI in dermatology is needed before trust can be established (3.87 ± 0.88).

Willingness to personally use AI for skin conditions (3.60 ± 0.97) and intention to use AI applications in the future (3.65 ± 0.94) were moderate, indicating generally favorable but cautious attitudes toward AI-assisted dermatological care.

### 3.3. Reliability and Internal Consistency

The attitude scale demonstrated excellent internal consistency (Cronbach’s α = 0.868; McDonald’s ω = 0.888). The mean overall AI attitude orientation score was 74.48 ± 10.20 (range 23–100).

Among subdomains, cultural considerations had the highest mean score (15.72 ± 2.63), followed by perceived benefits (14.97 ± 3.01) and trust (14.72 ± 2.39). Intention to use AI showed moderate scores (7.25 ± 1.78) with strong reliability (α = 0.849). Knowledge (10.86 ± 1.98) and concerns (10.96 ± 2.28) had comparatively lower mean scores and lower internal consistency (Table 3).

### 3.4. Factors Associated with AI Attitude Orientation and Perceived Importance

Age was significantly associated with overall AI attitude orientation scores (*p* = 0.009), with the highest mean scores observed among participants aged 18–24 years (75.96 ± 9.38). However, as post-hoc pairwise comparisons were not performed, specific between-group differences should be interpreted with caution. No significant differences were observed across gender, region, education, dermatology experience, or healthcare background.

Perceived importance scores were significantly higher among participants affiliated with healthcare (*p* = 0.004) and those with greater technological experience (*p* = 0.014).

Interest in technology demonstrated the strongest association with both AI attitude orientation and perceived importance scores (*p* < 0.001). Participants with very high interest reported the highest acceptance (76.76 ± 11.79) and importance (25.52 ± 5.42) scores, whereas those with very low interest reported the lowest scores.

Use of mobile health applications was not significantly associated with AI attitude orientation or perceived importance (*p* > 0.05) (Table 4).

### 3.5. Perceived Importance of AI Features

Privacy protection and data security (56.4%) and diagnostic accuracy (55.8%) were the most frequently rated “very important” features. Decision interpretability (47.2%) and cultural compatibility (49.3%) were also highly prioritized. Ease of use (42.8%) and cost (40.0%) were somewhat less frequently rated as very important but remained valued overall.

**Figure 1:**
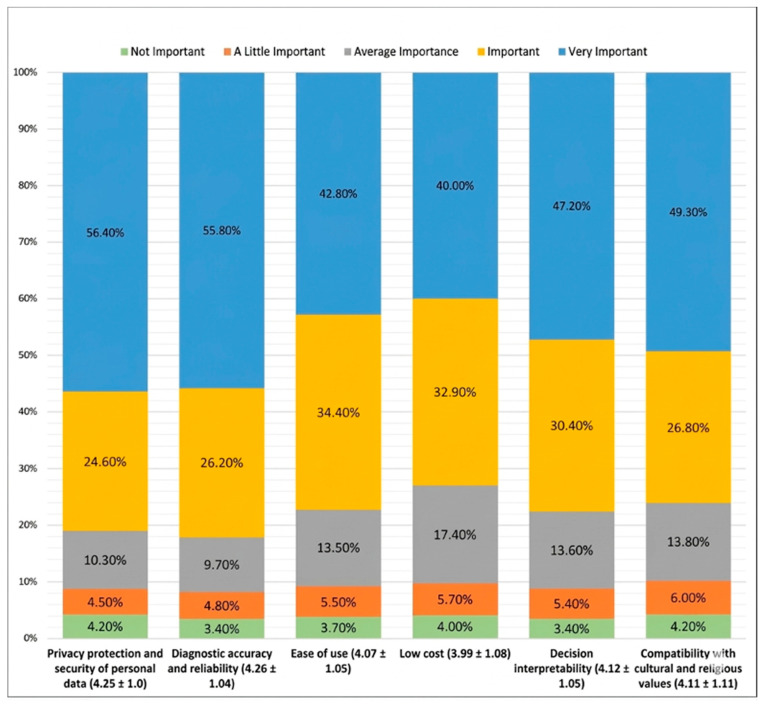
Perceived importance of dermatological AI system features (N = 668). Responses were rated on a 5-point scale (1 = not important to 5 = very important). Bars represent response distribution. Mean scores ± SD are shown below each feature.

The importance scale demonstrated excellent internal consistency (Cronbach’s α = 0.951) (Figure 1).

### 3.6. Predictors of High Intention to Use AI

Multivariate logistic regression identified no significant independent predictors of high intention to use dermatological AI (Table 5). Age, gender, geographic region, educational level, dermatologist visit history, healthcare affiliation, technology experience, interest in technology, and use of mobile health applications were not significantly associated with high intention to use AI (all *p* > 0.05).

## 4. Discussion

### 4.1. Principal Findings

This nationwide cross-sectional study suggests that Saudi adults generally hold favorable but cautious attitudes toward artificial intelligence (AI) in dermatology. Participants expressed interest in the potential benefits of AI while simultaneously emphasizing the importance of physician oversight, transparency, privacy protection, and clinical accountability. Participants indicated a clear preference for physician-supervised AI, while support for fully autonomous systems was limited. Respondents acknowledged potential benefits, particularly improved access to care and cost efficiency; however, trust in independent AI-driven decision-making remained moderate.

A consistent pattern across responses was the prioritization of privacy protection, diagnostic accuracy, and decision transparency. These attributes function as core determinants of trust rather than optional system features. Collectively, the findings indicate that acceptance of dermatological AI is influenced not only by perceived utility but also by alignment with expectations of safety, accountability, and clinical oversight.

Age and technological engagement were the only variables significantly associated with overall AI attitude orientation scores. Younger participants and those with greater interest in technology demonstrated higher acceptance, while other demographic and healthcare-related factors showed no independent association.

These findings are based on a general population sample without prior clinical AI exposure, providing a pre-adoption baseline critical for informing implementation strategies. The findings suggest cautious receptiveness toward AI-assisted dermatological care rather than unconditional acceptance. Public willingness to engage with dermatological AI appears closely linked to the presence of appropriate governance, transparency, and physician involvement.

### 4.2. Comparison with Previous Studies

The observed pattern of conditional acceptance is consistent with prior literature indicating that public trust in healthcare AI depends on its role as an adjunct to, rather than a replacement for, clinicians [15,16,17]. Across settings, physician involvement functions as a central mechanism through which patients interpret safety, accountability, and reliability in AI-supported care [18,19].

The strong preference for supervised models likely reflects a broader trust framework in which clinical authority reduces uncertainty inherent in algorithmic decision-making. This interpretation is supported by the emphasis on explainability and physician override in cases of diagnostic disagreement, indicating that acceptance is closely linked to perceived controllability of AI systems.

The association between younger age, technological interest, and higher AI attitude orientation scores aligns with established models of digital health adoption [16,18]. Familiarity with emerging technologies may reduce perceived risk and enhance perceived benefit. In contrast, the absence of increased acceptance among healthcare-affiliated participants, despite their higher prioritization of system features, suggests a more critical appraisal of AI. This finding is consistent with prior reports of cautious attitudes among clinicians [20]. Privacy concerns and emphasis on diagnostic reliability are well documented in studies of healthcare AI adoption [15,21], reinforcing that technical performance alone is insufficient for acceptance. Trust is mediated by governance, transparency, and risk perception.

The present findings are further contextualized by recent comparator studies. Wu et al. reported that dermatology patients in a US academic setting preferred combined AI and physician decision-making, deferring to clinician judgment when disagreement occurred [6]. Similarly, Ashraf et al. documented a modestly positive orientation without significant sociodemographic predictors in a Pakistani outpatient cohort [7]. The convergence of this conditional acceptance pattern across culturally distinct populations, including the United States, Pakistan, and Saudi Arabia, suggests that physician-supervised AI represents a cross-culturally robust governance preference. This is further supported by the narrative review by McRae et al., which identified physician oversight as the most consistent prerequisite for acceptance across 48 studies [5].

The absence of significant independent demographic predictors warrants interpretation beyond methodological considerations. Similar null findings have been reported in both Pakistani and US cohorts [7,8], suggesting that AI acceptance in dermatology is not strongly stratified by demographic factors. Favorable attitudes toward physician-supervised AI appear broadly distributed across populations, supporting population-level rather than demographically targeted implementation strategies. In contrast, the consistent association between technology familiarity and acceptance across studies highlights AI literacy as a potentially modifiable determinant of adoption.

Comparison with clinician perspectives provides important cross-stakeholder context. The international survey by Karampinis et al. identified regulatory uncertainty and medico-legal accountability, rather than technical performance, as the primary barriers to AI adoption among dermatologists [4]. Similarly, Al-Ali et al. reported openness to AI among Saudi clinicians, alongside concerns regarding professional autonomy [12]. These concern profiles differ from those of the public, who prioritized privacy, transparency, and data security. This divergence reflects distinct orientations: patients emphasize personal data governance, while clinicians emphasize institutional accountability. Both groups, however, converge on the importance of physician oversight, providing a shared foundation for implementation.

### 4.3. Implications for AI Implementation in Saudi Arabia

These findings have several implications for the implementation of AI in Saudi dermatology. First, they support a physician-centered integration model in which AI serves as a decision-support tool embedded within clinical workflows, rather than as an autonomous diagnostic system.

Second, the prominence of privacy and transparency concerns underscores the need for robust data governance frameworks and explainable AI systems. Addressing these issues is as critical as optimizing diagnostic performance to foster public trust and ensure sustained adoption. Furthermore, the lack of consistent sociodemographic predictors of adoption suggests that acceptance is not strongly segmented by demographic factors, underscoring the importance of system-level attributes such as transparency, accountability, and perceived safety as primary determinants of public engagement.

Third, the importance assigned to cultural compatibility indicates that implementation strategies should incorporate context-specific design considerations to ensure alignment with societal values and patient expectations [22]. This is particularly relevant in healthcare systems undergoing rapid digital transformation, where trust must be established alongside technological deployment.

Fourth, as AI diagnostic tools are introduced, it will be essential to ensure that training datasets adequately represent the range of Fitzpatrick skin types within the Saudi population. This approach is necessary to maintain equitable performance and prevent bias, as highlighted in prior studies [8].

Finally, the convergence of public preferences observed in this study with previously reported clinician attitudes toward physician-supervised AI provides a stable foundation for implementation. This alignment suggests that a collaborative governance model can be operationalized with relatively limited resistance from key stakeholder groups.

### 4.4. Limitations

Several limitations should be considered when interpreting these findings. First, the cross-sectional design restricts causal inference. More importantly, the use of convenience and snowball sampling through social media platforms may have substantially limited the representativeness of the study population. Recruitment through WhatsApp, Snapchat, X/Twitter, and Telegram likely favored individuals who were younger, more technologically engaged, and more familiar with digital tools, potentially resulting in more favorable attitudes toward AI than might be observed in the general population. This concern is reflected in the age distribution of the sample, where nearly half of participants were aged 18–24 years, while only a small proportion were aged 55 years or older. Because older adults represent an important group of dermatology service users and may hold different perspectives regarding digital health technologies, the findings should be interpreted with caution and should not be considered fully representative of the broader Saudi adult population. Consequently, the absence of demographic effects in the multivariable analysis should also be interpreted cautiously.

Second, although the 20-item attitudinal instrument demonstrated strong overall internal consistency, certain subdomains exhibited lower reliability. This likely results from the brevity of the scales and the multidimensional nature of AI-related perceptions. As a result, subdomain scores were used for descriptive profiling rather than as independent latent constructs. Furthermore, because concern-oriented items were not reverse-coded, the total score represents a holistic attitudinal orientation rather than a unidimensional measure of acceptance. Comparisons with studies employing reverse-coded concern items should be interpreted in this context.

Finally, the use of self-reported survey data captures attitudes in an abstract, pre-adoption context and may not directly correspond to real-world behavior or clinical decision-making after exposure to operational AI systems. Future research should incorporate longitudinal designs, behavioral outcome measures, and qualitative approaches to more effectively characterize how trust, perceived utility, and adoption evolve during real-world implementation.

## 5. Conclusions

This study provides a baseline assessment of public attitudes toward AI in dermatology in Saudi Arabia, demonstrating generally favorable yet cautious attitudes toward AI in dermatology that are consistent with international findings. Multivariable analysis showed no consistent sociodemographic predictors of adoption, suggesting that acceptance is not strongly demographically stratified but is instead shaped by system-level factors. Public willingness to adopt AI appears to depend on the perceived safety, transparency, and clinical governance of these systems. These findings have several potential implications for future implementation efforts. In settings where dermatological AI is introduced, physician-supervised decision-support models, explainable system design, robust data governance frameworks, and culturally responsive adaptation may help address the priorities identified by participants in this study. While the present findings do not directly evaluate implementation outcomes, they provide insight into factors that may influence public trust and acceptance during future deployment of AI-enabled dermatological services.

## Figures and Tables

**Table 1:** Participant sociodemographic and clinical characteristics (N = 668).

Variable	Category	N	%
Age	18–24 years	303	45.4
	25–34 years	187	28.0
	35–44 years	92	13.8
	45–54 years	65	9.7
	55 years and older	21	3.1
Gender	Female	408	61.1
	Male	260	38.9
Region	Central Region	295	44.2
	Eastern Region	189	28.3
	Northern Region	39	5.8
	Southern Region	42	6.3
	Western Region	103	15.4
Educational level	High school or less	165	24.7
	Diploma	83	12.4
	Bachelor’s	371	55.5
	Postgraduate (Master’s/PhD)	49	7.3
Dermatologist experience	Never visited	179	26.8
	Visited once	166	24.9
	Visited several times	247	37.0
	Regular visits (>once/year)	76	11.4
Works/studies in healthcare	No	488	73.1
	Yes	180	26.9
Technology experience	Beginner user	83	12.4
	Average user	473	70.8
	Expert/Professional	112	16.8
Interest in new technologies	Very low	17	2.5
	Low	19	2.8
	Average	255	38.2
	High	224	33.5
	Very high	153	22.9
Uses smart health apps	No	296	44.3
	Yes	372	55.7

**Table 2:** Distribution of responses and mean scores for attitudes toward artificial intelligence in dermatology (N = 668).

Statement	Strongly Disagree	Disagree	Neutral	Agree	Strongly Agree	Mean ± SD
I have knowledge about AI applications in dermatology	57 (8.5%)	103 (15.4%)	288 (43.1%)	169 (25.3%)	51 (7.6%)	3.08 ± 1.02
I need more information about AI in dermatology before trusting it	15 (2.2%)	29 (4.3%)	133 (19.9%)	340 (50.9%)	151 (22.6%)	3.87 ± 0.88
I have a positive outlook on AI in healthcare	16 (2.4%)	29 (4.3%)	140 (21.0%)	300 (44.9%)	183 (27.4%)	3.91 ± 0.93
I trust dermatologists’ expertise	8 (1.2%)	22 (3.3%)	133 (19.9%)	333 (49.9%)	172 (25.7%)	3.96 ± 0.83
I trust AI applications for skin diagnosis	30 (4.5%)	98 (14.7%)	275 (41.2%)	200 (29.9%)	65 (9.7%)	3.26 ± 0.97
I prefer physician diagnosis over AI when they disagree †	11 (1.6%)	14 (2.1%)	110 (16.5%)	287 (43.0%)	246 (36.8%)	4.11 ± 0.86
I feel comfortable with AI for simple skin conditions	32 (4.8%)	102 (15.3%)	199 (29.8%)	244 (36.5%)	91 (13.6%)	3.39 ± 1.05
I prefer AI-assisted physician diagnosis	32 (4.8%)	66 (9.9%)	190 (28.4%)	252 (37.7%)	128 (19.2%)	3.57 ± 1.06
AI can help in early detection of serious skin diseases	15 (2.2%)	47 (7.0%)	218 (32.6%)	250 (37.4%)	138 (20.7%)	3.67 ± 0.95
AI can reduce healthcare costs	12 (1.8%)	25 (3.7%)	178 (26.6%)	314 (47.0%)	139 (20.8%)	3.81 ± 0.86
AI benefits patients in remote areas	18 (2.7%)	27 (4.0%)	136 (20.4%)	295 (44.2%)	192 (28.7%)	3.92 ± 0.94
AI needs medical supervision †	14 (2.1%)	8 (1.2%)	81 (12.1%)	248 (37.1%)	317 (47.5%)	4.27 ± 0.87
I trust AI in emergency situations	39 (5.8%)	87 (13.0%)	217 (32.5%)	248 (37.1%)	77 (11.5%)	3.35 ± 1.03
AI decisions should be explainable †	7 (1.0%)	14 (2.1%)	107 (16.0%)	301 (45.1%)	239 (35.8%)	4.12 ± 0.82
AI should respect cultural values	11 (1.6%)	28 (4.2%)	131 (19.6%)	297 (44.5%)	201 (30.1%)	3.97 ± 0.90
I have privacy concerns about AI ‡	19 (2.8%)	50 (7.5%)	160 (24.0%)	258 (38.6%)	181 (27.1%)	3.80 ± 1.01
I worry AI might delay proper treatment ‡	14 (2.1%)	25 (3.7%)	140 (21.0%)	299 (44.8%)	190 (28.4%)	3.94 ± 0.91
I’m concerned about AI bias ‡	62 (9.3%)	107 (16.0%)	217 (32.5%)	180 (26.9%)	102 (15.3%)	3.23 ± 1.16
I’m personally willing to use AI for skin conditions	25 (3.7%)	51 (7.6%)	196 (29.3%)	288 (43.1%)	108 (16.2%)	3.60 ± 0.97
I would use AI apps in the future	22 (3.3%)	45 (6.7%)	189 (28.3%)	303 (45.4%)	109 (16.3%)	3.65 ± 0.94

Note: † Items reflecting governance and oversight preferences. ‡ Items reflecting concern or caution. High agreement with these items indicates conditional acceptance or apprehension rather than endorsement of AI autonomy.

**Table 3:** Subdomain scores and internal consistency of the attitude scale.

Subdomain	Items	Mean	SD	Min	Max	Cronbach’s Alpha (95% CI)	McDonald’s Omega
Overall AI attitude orientation score	20	74.48	10.20	23.00	100.00	0.868 (0.818–0.918)	0.888
Knowledge	3	10.86	1.98	3.00	15.00	0.465 (0.415–0.515) *	0.485
Trust	4	14.72	2.39	4.00	20.00	0.514 (0.464–0.564) *	0.534
Benefits	4	14.97	3.01	4.00	20.00	0.792 (0.742–0.842)	0.812
Cultural	4	15.72	2.63	4.00	20.00	0.690 (0.640–0.740)	0.710
Concerns	3	10.96	2.28	3.00	15.00	0.575 (0.525–0.625) *	0.595
Intention	2	7.25	1.78	2.00	10.00	0.849 (0.799–0.899)	0.869

Note: * Cronbach’s α < 0.60, reflecting item brevity and multidimensionality. Scores interpreted descriptively only.

**Table 4:** Association of participant characteristics with overall AI attitude orientation and perceived importance scores.

Variable	Category	N	AI Attitude Orientation Score		Importance	
			Mean ± SD	*p*-Value	Mean ± SD	*p*-Value
Age	18–24	303	75.96 ± 9.38	0.009	25.40 ± 5.50	0.107
	25–34	187	72.88 ± 11.59		24.56 ± 5.89	
	35–44	92	73.28 ± 10.53		24.04 ± 5.35	
	45–54	65	73.40 ± 8.59		24.38 ± 6.44	
	≥55	21	75.86 ± 9.07		23.00 ± 7.13	
Gender	Female	408	74.52 ± 10.08	0.882	24.71 ± 6.02	0.607
	Male	260	74.40 ± 10.40		24.95 ± 5.34	
Region	Central	295	74.13 ± 10.43	0.891	24.65 ± 5.88	0.782
	Eastern	189	74.93 ± 10.35		24.67 ± 6.02	
	Northern	39	75.10 ± 12.95		24.77 ± 5.86	
	Southern	42	75.12 ± 8.64		25.79 ± 4.51	
	Western	103	74.15 ± 8.70		25.08 ± 5.36	
Educational level	High school or less	165	74.57 ± 9.65	0.337	24.22 ± 6.32	0.058
	Diploma	83	73.22 ± 11.99		24.71 ± 5.69	
	Bachelor’s degree	371	74.94 ± 9.37		25.28 ± 5.23	
	Postgraduate (Master’s/PhD)	49	72.80 ± 14.05		23.33 ± 7.26	
Dermatologist experience	Never visited	179	73.55 ± 9.93	0.365	24.80 ± 5.87	0.134
	Visited once	166	74.39 ± 10.00		24.14 ± 6.03	
	Visited several times	247	74.77 ± 9.79		25.41 ± 5.40	
	Regular visits	76	75.91 ± 12.34		24.26 ± 5.94	
Works/studies in healthcare	No	488	74.44 ± 10.42	0.871	24.42 ± 5.93	0.004
	Yes	180	74.58 ± 9.59		25.84 ± 5.14	
Technology experience	Beginner user	83	73.16 ± 11.97	0.452	23.14 ± 7.16	0.014
	Average user	473	74.66 ± 9.41		25.14 ± 5.36	
	Expert/Professional	112	74.67 ± 11.87		24.63 ± 6.06	
Interest in technology	Very low	17	67.71 ± 11.90	<0.001	20.06 ± 8.52	<0.001
	Low	19	66.11 ± 12.22		22.16 ± 8.54	
	Average	255	73.56 ± 9.94		24.18 ± 5.84	
	High	224	75.18 ± 8.20		25.61 ± 5.04	
	Very high	153	76.76 ± 11.79		25.52 ± 5.42	
Uses smart health apps	No	296	73.75 ± 10.02	0.102	24.61 ± 5.82	0.445
	Yes	372	75.05 ± 10.31		24.95 ± 5.72	

**Table 5:** Multivariate logistic regression analysis of factors associated with high intention to use dermatological AI.

Independent Variable	Category	OR (95% CI)	*p*-Value
Age	18–24	1.033 (0.708–1.508)	0.865
	25–34	1.358 (0.829–2.224)	0.225
	35–44	0.788 (0.477–1.301)	0.351
	45–54	1.449 (0.440–4.771)	0.542
	≥55 (Reference)	N/A	N/A
Gender	Male	0.881 (0.635–1.220)	0.445
	Female (Reference)	N/A	N/A
Region	Central	1.029 (0.712–1.486)	0.880
	Eastern	0.879 (0.381–2.030)	0.763
	Northern	1.242 (0.650–2.374)	0.512
	Southern	1.077 (0.677–1.714)	0.754
	Western (Reference)	N/A	N/A
Educational level	Diploma	1.009 (0.636–1.601)	0.970
	Bachelor’s degree	0.952 (0.642–1.411)	0.805
	Postgraduate (Master’s/PhD)	1.251 (0.668–2.346)	0.484
	High school or less (Reference)	N/A	N/A
Dermatologist experience	Visited once	0.981 (0.641–1.499)	0.928
	Visited several times	0.613 (0.342–1.097)	0.099
	Regular visits	0.947 (0.638–1.406)	0.787
	Never visited (Reference)	N/A	N/A
Works/studies in healthcare	Yes	1.104 (0.780–1.562)	0.576
	No (Reference)	N/A	N/A
Technology experience	Average user	1.460 (0.890–2.392)	0.134
	Expert/Professional	0.992 (0.659–1.493)	0.969
	Beginner user (Reference)	N/A	N/A
Interest in technology	Low	0.771 (0.268–2.218)	0.630
	Average	1.053 (0.719–1.540)	0.792
	High	1.034 (0.679–1.574)	0.877
	Very high	0.413 (0.150–1.132)	0.086
	Very low (Reference)	N/A	N/A
Uses smart health apps	Yes	1.103 (0.808–1.507)	0.537
	No (Reference)	N/A	N/A

Note: Abbreviations: OR, odds ratio; CI, confidence interval; N/A, not applicable (reference category).

## Data Availability

The data presented in this study are available on request from the corresponding author due to privacy restrictions.
